# Association between *Mycobacterium tuberculosis* Complex Phylogenetic Lineage and Acquired Drug Resistance

**DOI:** 10.1371/journal.pone.0083006

**Published:** 2013-12-23

**Authors:** Courtney M. Yuen, Ekaterina V. Kurbatova, Eleanor S. Click, J. Sean Cavanaugh, J. Peter Cegielski

**Affiliations:** Division of Tuberculosis Elimination, National Center for HIV/AIDS, Viral Hepatitis, STD, and TB Prevention, U.S. Centers for Disease Control and Prevention, Atlanta, Georgia, United States of America; McGill University, Canada

## Abstract

**Background:**

Development of resistance to antituberculosis drugs during treatment (i.e., acquired resistance) can lead to emergence of resistant strains and consequent poor clinical outcomes. However, it is unknown whether *Mycobacterium tuberculosis* complex species and lineage affects the likelihood of acquired resistance.

**Methods:**

We analyzed data from the U.S. National Tuberculosis Surveillance System and National Tuberculosis Genotyping Service for tuberculosis cases during 2004–2011 with assigned species and lineage and both initial and final drug susceptibility test results. We determined univariate associations between species and lineage of *Mycobacterium tuberculosis* complex bacteria and acquired resistance to isoniazid, rifamycins, fluoroquinolones, and second-line injectables. We used Poisson regression with backward elimination to generate multivariable models for acquired resistance to isoniazid and rifamycins.

**Results:**

*M. bovis* was independently associated with acquired resistance to isoniazid (adjusted prevalence ratio = 8.46, 95% CI 2.96–24.14) adjusting for HIV status, and with acquired resistance to rifamycins (adjusted prevalence ratio = 4.53, 95% CI 1.29–15.90) adjusting for homelessness, HIV status, initial resistance to isoniazid, site of disease, and administration of therapy. East Asian lineage was associated with acquired resistance to fluoroquinolones (prevalence ratio = 6.10, 95% CI 1.56–23.83).

**Conclusions:**

We found an association between mycobacterial species and lineage and acquired drug resistance using U.S. surveillance data. Prospective clinical studies are needed to determine the clinical significance of these findings, including whether rapid genotyping of isolates at the outset of treatment may benefit patient management.

## Introduction

The development of resistance to antituberculosis drugs during treatment, known as acquired resistance, can lead to the emergence of resistant strains and consequent poor clinical outcomes [1]–[3]. Acquired resistance is associated with improper use of antibiotics, inadequate regimens, or incomplete treatment [4]–[6]. However, it is not known if genetic bacterial strain differences are associated with the likelihood of acquired resistance.

The *Mycobacterium tuberculosis* complex (MTBC) comprises several species, of which *M. tuberculosis*, *M. bovis, and M. africanum* are most commonly associated with tuberculosis in humans. Of these, *M. bovis* is unique in that it is intrinsically resistant to pyrazinamide, one of the first-line drugs used to treat tuberculosis [7]. Four main phylogenetic lineages of *M. tuberculosis* (Indo-Oceanic, East Asian, East African-Indian, and Euro-American) and two lineages of *M. africanum* (West African 1 and 2) have been identified [8]. Studies from a variety of settings have reported associations between East Asian lineage or its dominant member, the Beijing genotype, and drug resistance [9]–[13]. However, these epidemiologic studies have not looked specifically at acquired resistance documented by repeated drug susceptibility testing during the course of tuberculosis treatment. Thus, these associations may reflect an increased risk of being infected with a strain that is already drug-resistant rather than an increased risk of developing resistance during treatment.

Because of the clinical and public health implications of developing resistance during treatment, we sought to determine whether MTBC species or lineage was associated with acquired resistance to four major classes of antituberculosis drugs using data from the U.S. National Tuberculosis Surveillance System (NTSS) and National Tuberculosis Genotyping Service (NTGS).

## Methods

### Case Inclusion

We analyzed NTSS data for new (i.e., occurring in a patient without prior history of tuberculosis) tuberculosis cases with baseline positive cultures from the 50 states and the District of Columbia during 2004–2011 for which a species or lineage was assigned, and for which drug susceptibility test (DST) results were available. NTSS collects sociodemographic and clinical information, including DST results, on all tuberculosis cases in the United States. DSTs for first-line drugs on the initial positive culture are routine in the United States, but repeated DSTs and DSTs on second-line drugs are performed only when indicated. DST results for the first isolate tested (i.e., initial DST results) are reported to NTSS; if repeated DST is performed, results for the last isolate on which DST was performed (i.e., final DST results) are reported to NTSS. Since 2004, NTGS has routinely performed genotyping on culture-positive cases and assigned lineage based on spoligotype and 12-locus mycobacterial interspersed repetitive unit variable number of tandem repeat [14]. The rules used for classification of lineage have been shown produce results consistent with lineage assignment based on long-sequence polymorphisms [14]–[16]. Data from NTGS and NTSS were linked to identify cases with assigned lineage.

We analyzed cases with and without acquired resistance to isoniazid, rifamycins (rifampin, rifabutin, and rifapentine), second-line injectables (amikacin, kanamycin, and capreomycin, referred to collectively from here as “injectables”), and fluoroquinolones (ciprofloxacin, levofloxacin, moxifloxacin, ofloxacin, and drugs classified as “other quinolone” in NTSS). For each drug class, we restricted analysis to cases with initial susceptibility to all tested members of the class and a final DST result for at least one member. Acquired resistance to a drug was defined as resistance at the final DST to a drug to which the case’s first isolate had been susceptible at the initial DST. Acquired resistance to a drug class was defined as acquired resistance to any of the drugs in the class. No acquired resistance to a drug was defined as susceptibility to that drug at both initial and final DST. No acquired resistance to a drug class was defined as susceptibility at both initial and final DST to at least one member of the class, and susceptibility at final DST to all tested members of the class.

### Statistical Analysis

We determined characteristics associated with acquired resistance to isoniazid, rifamycins, injectables, and fluoroquinolones by calculating prevalence ratios (PR) with 95% confidence intervals (CI). A 95% CI excluding the null value (i.e., PR = 1.0) was considered statistically significant. We used multivariable Poisson regression to assess factors independently associated with acquired resistance to isoniazid and rifamycins. The initial multivariable model included species and lineage as well as variables with *P*<0.10 in univariate analysis and potential confounders based on biological plausibility or known epidemiologic association. We assessed confounding in the initial model and used backward elimination to determine variables included in the final model to obtain better precision around point estimates. To test the robustness of the multivariable model, we carried out sensitivity analyses using subsets of included cases. In one analysis, we included only cases caused by *M. tuberculosis* (i.e., excluding those caused by *M. bovis* and *M. africanum*); in another, we included only cases with initial susceptibility to isoniazid, rifampin, and ethambutol. We did not perform multivariable analysis for injectables and fluoroquinolones because of the small number of cases with documented acquired resistance to these drug classes. All statistical analysis was performed using SAS version 9.3 (SAS Institute Inc., Cary, NC).

### Ethics Statement and Public Availability of Data

All data were collected and analyzed as part of routine public health surveillance. The project was therefore determined not to be human subjects research by the U.S. Centers for Disease Control and Prevention (CDC) and did not require approval by an institutional review board.

Researchers may apply to analyze NTSS and NTGS data at CDC headquarters according to established procedures. In addition, a publicly available version of NTSS data is available at https://wonder.cdc.gov/tb.html.

## Results

**Figure 1:**
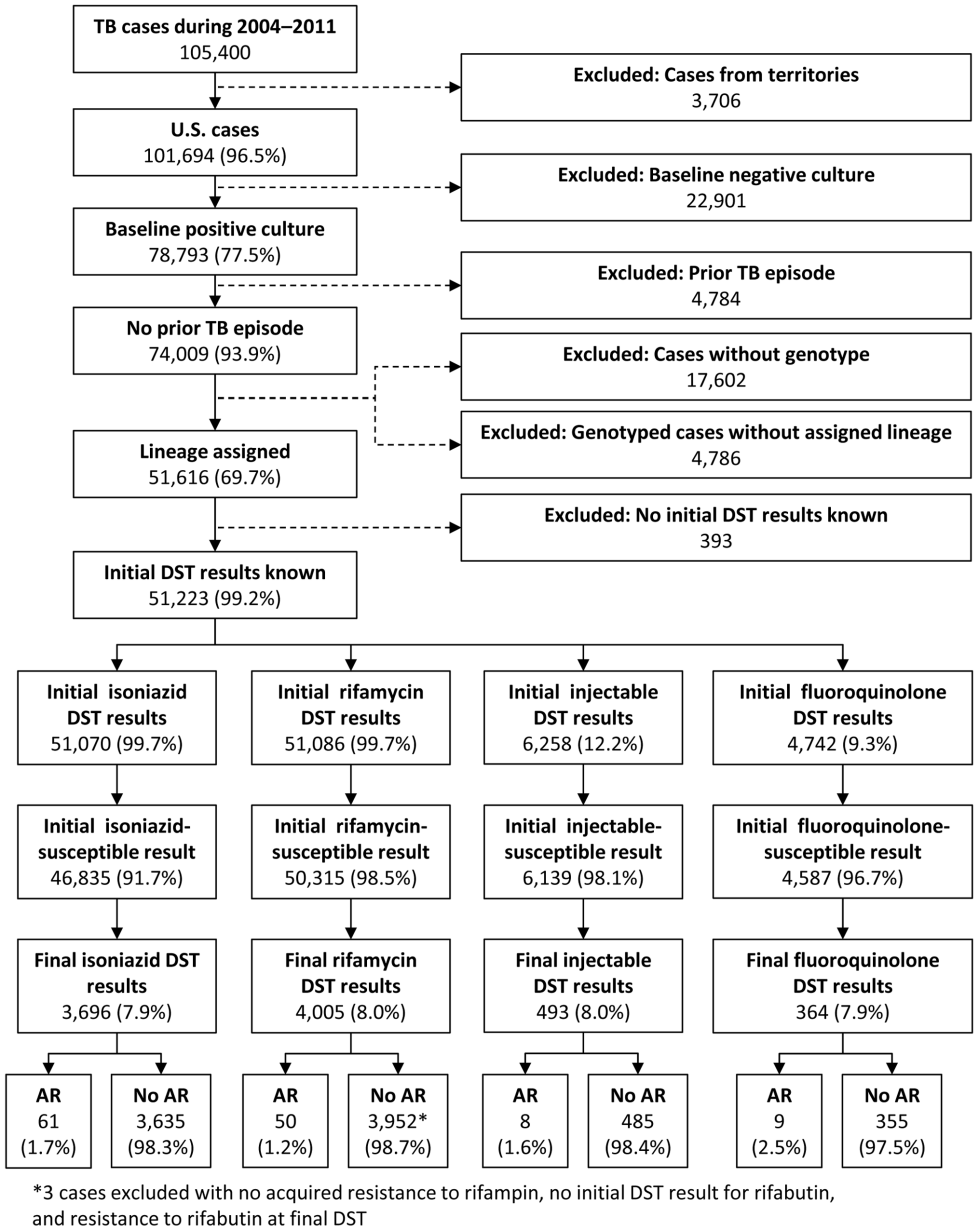
Selection of cases included in analysis. Abbreviations: TB = tuberculosis, DST = drug susceptibility test, AR = acquired resistance.

Among 105,400 tuberculosis cases counted by NTSS during 2004–2011, 51,223 (48.6%) met our inclusion criteria (Figure 1). Among this subset of cases, initial DST for isoniazid and at least one rifamycin were almost universal (>99%). In contrast, 6,258 (12.2%) and 4,742 (9.3%) had an initial DST result for at least one injectable or fluoroquinolone, respectively. For all four drug classes, the majority of cases (>90%) with initial DST results showed susceptibility at initial DST. For all four drug classes, approximately 8% of cases initially susceptible to the drug class had a final DST result. Out of cases with final DST results, the proportion of cases with acquired resistance ranged from 1.2% for rifamycins to 2.5% for fluoroquinolones.

Species and phylogenetic lineage was associated with acquired resistance to isoniazid, rifamycins, injectables, and fluoroquinolones (Table 1). Because the numbers of cases with final DST results for injectables and fluoroquinolones was small, we calculated only crude PRs for these drug classes. *M. bovis* was associated with acquired resistance to both isoniazid (adjusted prevalence ratio [aPR] = 8.46, 95% CI 2.96–24.14) and rifamycins (aPR = 4.53, 95% CI 1.29–15.90). East Asian lineage was associated with acquired resistance to fluoroquinolones (PR = 6.10, 95% CI 1.56–23.83).

**Table 1:** Associations between *M. tuberculosis* complex lineage and acquired resistance to isoniazid, rifamycins, second-line injectables, and fluoroquinolones.

	Acquired resistance to isoniazid		Acquired resistance to rifamycins		Acquired resistance to injectables	Acquired resistance to fluoroquinolones
Species, lineage	Crudeprevalenceratio (95% CI)	Adjusted*prevalenceratio (95%CI)	Crudeprevalenceratio (95% CI)	Adjusted†prevalenceratio (95%CI)	Crudeprevalenceratio (95% CI)	Crudeprevalenceratio (95% CI)
*M. africanum*	–	–	–	–	–	–
*M. bovis*	**7.03 (2.66–18.56)**	**8.46 (2.96–24.14)**	**6.61 (2.12–20.65)**	**4.53 (1.29–15.90)**	–	–
*M. tuberculosis,* EastAfrican-Indian	0.46 (0.06–3.33)	0.48 (0.07–3.49)	0.56 (0.08–4.05)	1.09 (0.14–8.19)	3.64 (0.42–31.27)	–
*M. tuberculosis,* East Asian	1.06 (0.52–2.15)	1.10 (0.45–2.90)	1.31 (0.63–2.72)	1.84 (0.86–3.93)	2.59 (0.59–11.38)	**6.10 (1.56–23.83)**
*M. tuberculosis,* Euro-American	Reference	Reference	Reference	Reference	Reference	Reference
*M. tuberculosis,* Indo-Oceanic	1.02 (0.41–2.56)	1.14 (0.45–2.90)	0.94 (0.34–2.65)	1.46 (0.49–4.31)	–	–

* Adjusted for HIV status.

† Adjusted for homelessness, HIV status, initial resistance to isoniazid, site of disease, and administration of therapy.

Abbreviations: CI = confidence interval, – = no prevalence ratio calculated because no cases had acquired resistance.

Sociodemographic and clinical factors showed no statistically significant associations with acquired resistance to isoniazid (Table 2). In contrast, acquired resistance to rifamycins was associated with older age groups (45–64 and ≥65 years), homelessness, injecting drug use, HIV infection, initial resistance to isoniazid, initial resistance to ethambutol, combined pulmonary and extrapulmonary site of disease (including miliary tuberculosis), and self-administered therapy (Table 3).

**Table 2:** Sociodemographic and clinical factors associated with acquired resistance to isoniazid (N = 3,696).

		Included cases	Acquired resistancen (% of included)		Crude PR (95% CI)	
Species, lineage	*M. africanum*	16	0	(0.0)	–	
	*M. bovis* *M. tuberculosis*	36	4	(11.1)	**7.03**	**(2.66–18.56)**
	*M. tuberculosis*, East African-Indian	137	1	(0.7)	0.46	(0.06–3.33)
	*M. tuberculosis*, East Asian	541	9	(1.7)	1.05	(0.52–2.15)
	*M. tuberculosis*, Euro-American	2,656	42	(1.6)	Reference	
	*M. tuberculosis*, Indo-Oceanic	310	5	(1.6)	1.02	(0.41–2.56)
Sex	Female	1,068	23	(1.7)	1.03	(0.60–1.78)
	Male	2,628	51	(1.6)	Reference	
Age	0–24	363	6	(1.4)	0.80	(0.31–2.11)
	25–44	1,284	28	(1.7)	Reference	
	45–64	1,407	30	(1.9)	1.12	(0.64–1.96)
	≥65	642	10	(1.1)	0.64	(0.27–1.48)
Race and ethnicity	Hispanic	1,123	24	(1.9)	1.18	(0.59–2.34)
	American Indian	28	1	(3.6)	2.25	(0.30–16.58)
	Asian	643	9	(1.2)	0.78	(0.33–1.88)
	Non-Hispanic Black	1,063	18	(1.7)	1.07	(0.53–2.34)
	Non-Hispanic White	818	13	(1.6)	Reference	
Country of birth	Foreign-born	1,864	32	(1.7)	0.91	(0.58–1.42)
	U.S.-born	1,829	29	(1.6)	Reference	
Region of birth*	Africa	188	1	(0.5)	0.34	(0.05–2.45)
	Americas (other than United States)	919	20	(2.2)	1.37	(0.78–2.41)
	Eastern Mediterranean	51	1	(2.0)	1.24	(0.17–8.90)
	Europe	74	2	(2.7)	1.70	(0.41–7.01)
	South-East Asia	169	2	(1.2)	0.75	(0.18–3.10)
	Western Pacific	458	6	(1.3)	0.83	(0.35–1.98)
	United States	1,829	29	(1.6)	Reference	
In correctional facility at diagnosis	Yes	133	2	(1.5)	0.91	(0.22–3.67)
	No	3,559	59	(1.7)	Reference	
Homeless	Yes	353	2	(0.6)	0.32	(0.08–1.30)
	No	3,328	59	(1.8)	Reference	
Injecting drug use	Yes	97	2	(2.1)	1.25	(0.31–5.04)
	No	3,572	59	(1.7)	Reference	
Non-injecting drug use	Yes	430	8	(1.9)	1.14	(0.44–1.90)
	No	3,235	53	(1.6)	Reference	
Excess alcohol use	Yes	870	10	(1.1)	0.73	(0.54–2.37)
	No	2,804	51	(1.8)	Reference	
HIV status	Positive	257	4	(1.6)	0.81	(0.29–2.23)
	Negative	2,497	48	(1.9)	Reference	
	Unknown	942	9	(1.0)	0.50	(0.24–1.01)
Initial DST result for rifamycins	Resistant to any	20	1	(5.0)	3.06	(0.45–21.01)
	Susceptible to all tested	3,672	60	(1.6)	Reference	
Initial DST result for pyrazinamide	Resistant	68	2	(2.9)	1.98	(0.49–8.01)
	Susceptible	2,619	39	(1.5)	Reference	
Initial DST result for ethambutol	Resistant	8	0	(0.0)	–	
	Susceptible	3,681	60	(1.6)	Reference	
Initial chest radiograph	Abnormal	3,440	55	(1.6)	0.78	(0.29–2.14)
	Normal	196	4	(2.0)	Reference	
Cavity seen on chest radiograph	Yes	1,598	27	(1.7)	1.07	(0.64–1.79)
	No	1,972	31	(1.6)	Reference	
Sputum microscopy result for AFB	Positive	2,764	44	(1.6)	0.83	(0.46–1.48)
	Negative	778	15	(1.9)	Reference	
Site of disease	Extrapulmonary only	179	1	(0.6)	0.35	(0.05–2.53)
	Pulmonary and extrapulmonary	364	10	(2.7)	1.73	(0.89–3.39)
	Pulmonary only	3,152	50	(1.6)	Reference	
Administration of therapy	DOT only	2,232	38	(1.7)	Reference	
	Self-administered and DOT	1,216	18	(1.5)	0.87	(0.50–1.52)
	Self-administered only	199	1	(0.5)	0.30	(0.04–2.14)

* Regions defined by World Health Organization [37].

Missing values not reported in table.

Abbreviations: PR = prevalence ratio, CI = confidence interval, DST = drug susceptibility test, AFB = acid-fast bacilli, DOT = directly observed therapy.

**Table 3:** Sociodemographic and clinical factors associated with acquired resistance to rifamycins (N = 4,005).

		Included cases	Acquired resistancen (% of included)		Crude PR (95% CI)	
Species, lineage	*M. africanum*	19	0	(0.0)	–	
	*M. bovis*	39	3	(7.7)	**6.61**	**(2.12–20.65)**
	*M. tuberculosis,* East African-Indian	154	1	(0.6)	0.56	(0.08–4.05)
	*M. tuberculosis,* East Asian	590	9	(1.5)	1.31	(0.63–2.72)
	*M. tuberculosis,* Euro-American	2,836	33	(1.2)	Reference	
	*M. tuberculosis,* Indo-Oceanic	364	4	(1.1)	0.94	(0.34–2.65)
Sex	Female	1,161	13	(1.1)	0.86	(0.46–1.61)
	Male	2,841	37	(1.3)	Reference	
Age	0–24	399	3	(0.8)	0.35	(0.11–1.14)
	25–44	1,399	30	(2.2)	Reference	
	45–64	1,531	15	(1.0)	**0.46**	**(0.25–0.85)**
	≥65	673	2	(0.3)	**0.14**	**(0.03–0.58)**
Race and ethnicity	Hispanic	1,222	17	(1.4)	1.72	(0.72–4.12)
	American Indian	30	0	(0.0)	–	
	Asian	748	8	(1.1)	1.32	(0.48–3.62)
	Non-Hispanic Black	1,113	18	(1.6)	2.00	(0.84–4.76)
	Non-Hispanic White	864	7	(0.8)	Reference	
Country of birth	Foreign-born	2,067	24	(1.2)	0.86	(0.50–1.50)
	U.S.-born	1,932	26	(1.3)	Reference	
Region of birth*	Africa	204	3	(1.5)	1.09	(0.33–3.58)
	Americas (other than United States)	993	12	(1.2)	0.90	(0.46–1.77)
	Eastern Mediterranean	58	1	(1.7)	1.28	(0.18–9.28)
	Europe	78	0	(0.0)	–	
	South-East Asia	191	2	(1.0)	0.78	(0.19–3.25)
	Western Pacific	538	6	(1.1)	0.83	(0.34–2.00)
	United States	1,932	26	(1.3)	Reference	
In correctional facility at diagnosis	Yes	150	4	(2.7)	2.23	(0.81–6.11)
	No	3,847	39	(1.2)	Reference	
Homeless	Yes	372	11	(3.0)	**2.74**	**(1.42–5.30)**
	No	3,613	39	(1.1)	Reference	
Injecting drug use	Yes	105	5	(4.8)	**4.09**	**(1.66–10.10)**
	No	3,867	45	(1.2)	Reference	
Non-injecting drug use	Yes	460	7	(1.5)	1.24	(0.56–2.74)
	No	3,508	43	(1.2)	Reference	
Excess alcohol use	Yes	933	14	(1.5)	1.27	(0.69–2.34)
	No	3,045	36	(1.2)	Reference	
HIV status	Positive	275	24	(8.7)	**12.46**	**(6.91–22.45)**
	Negative	2,712	19	(0.7)	Reference	
	Unknown	1,015	7	(0.7)	0.98	(0.42–2.23)
Initial DST result for isoniazid	Resistant	328	20	(6.1)	**7.45**	**(4.28–12.98)**
	Susceptible	3,667	30	(0.8)	Reference	
Initial DST result for pyrazinamide	Resistant	84	2	(2.4)	1.76	(0.43–7.16)
	Susceptible	2,878	39	(1.4)	Reference	
Initial DST result for ethambutol	Resistant	39	2	(5.1)	**4.22**	**(1.06–16.76)**
	Susceptible	3,952	48	(1.2)	Reference	
Initial chest radiograph	Abnormal	3,715	46	(1.2)	0.69	(0.25–1.91)
	Normal	224	4	(1.8)	Reference	
Cavity seen on chest radiograph	Yes	1,731	16	(0.9)	0.58	(0.32–1.05)
	No	2,136	34	(1.6)	Reference	
Sputum microscopy result for AFB	Positive	2,976	40	(1.3)	1.15	(0.58–2.29)
	Negative	854	10	(1.2)	Reference	
Site of disease	Extrapulmonary only	203	1	(0.5)	0.53	(0.07–3.82)
	Pulmonary and extrapulmonary	386	17	(4.4)	**4.70**	**(2.63–8.38)**
	Pulmonary only	3,412	32	(0.9)	Reference	
Administration of therapy	DOT	2,418	29	(1.2)	Reference	
	Self-administered and DOT	1,322	13	(1.0)	0.82	(0.43–1.57)
	Self-administered only	210	6	(2.9)	**2.38**	**(1.00–5.67)**

* Regions defined by World Health Organization [37].

Missing values not reported in table.

Abbreviations: PR = prevalence ratio, CI = confidence interval, DST = drug susceptibility test, AFB = acid-fast bacilli, DOT = directly observed therapy.

In multivariable analysis, acquired resistance to isoniazid was independently associated with *M. bovis* and with unknown HIV status (aPR = 0.46, 95% CI 0.23–0.95) (Table 4). Acquired resistance to rifamycins was associated with *M. bovis*, homelessness (aPR = 2.21, 95% CI 1.08–4.52), HIV infection (aPR = 8.89, 95% CI 4.43–17.85), initial resistance to isoniazid (aPR = 10.37, 95% CI 5.65–19.00), extrapulmonary site of disease (aPR = 2.31, 95% CI 1.17–4.58), and self-administered therapy (aPR = 2.52, 95% CI 1.01–6.30) (Table 4).

**Table 4:** Independent predictors of acquired resistance to isoniazid and rifamycins in multivariable regression analysis.

		Adjusted prevalence ratio for acquired resistance to isoniazid (95% CI)		Adjusted prevalence ratio for acquired resistance to rifamycins (95% CI)	
Species, lineage	*M. bovis*	**8.46**	**(2.96–24.14)**	**4.53**	**(1.29–15.90)**
	*M. tuberculosis*, East African Indian	0.48	(0.07–3.49)	1.09	(0.14–8.19)
	*M. tuberculosis*, East Asian	1.10	(0.53–2.25)	1.84	(0.86–3.93)
	*M. tuberculosis*, Euro American	Reference		Reference	
	*M. tuberculosis*, Indo Oceanic	1.14	(0.45–2.90)	1.46	(0.49–4.31)
Homeless	Yes	Not included in model		**2.21**	**(1.08–4.52)**
	No			Reference	
HIV status	Positive	0.67	(0.24–1.89)	**8.89**	**(4.43–17.85)**
	Negative	Reference		Reference	
	Unknown	**0.46**	**(0.23–0.95)**	0.75	(0.29–1.94)
Initial DST result for isoniazid	Resistant	Not included in model		**10.37**	**(5.65–19.00)**
	Susceptible			Reference	
Site of disease	Extrapulmonary only	Not included in model		**2.31**	**(1.17–4.58)**
	Pulmonary and extrapulmonary			0.44	(0.06–3.28)
	Pulmonary only			Reference	
Administration of therapy	DOT only	Not included in model		Reference	
	Self-administered and DOT			0.84	(0.44–1.63)
	Self-administered only			**2.52**	**(1.01–6.30)**

Abbreviation: CI = confidence interval, DST = drug susceptibility test, DOT = directly observed therapy.

We repeated univariate and multivariable analyses restricting cases to those caused by *M. tuberculosis* (data not shown). Similar associations with independent predictors other than *M. bovis* were observed for both acquired resistance to isoniazid and acquired resistance to rifamycins.

We also repeated univariate and multivariable analyses restricting cases to those with documented initial susceptibility to isoniazid, rifampin, and ethambutol (data not shown). Analysis of predictors associated with acquired resistance to isoniazid gave similar results. The association between *M. bovis* and acquired resistance to rifamycins lost statistical significance (aPR = 3.83, 95% CI 0.99–14.79), but independent associations with homelessness, HIV infection, and extrapulmonary site of disease were once again observed. We did not repeat analyses restricting cases to those with documented initial susceptibility to pyrazinamide because almost all *M. bovis* cases would have been excluded from analysis, as well as a substantial portion of other cases that lacked pyrazinamide DST results.

## Discussion

This large study among tuberculosis cases with repeated DST results suggests an association between species and lineage of MTBC bacteria and acquired resistance to antituberculosis drugs. We found *M. bovis* to be associated with acquired resistance to both isoniazid and rifamycins, and these associations were independent of other predictors of acquired resistance such as initial resistance to another first-line drug and HIV infection. In addition, our results suggest an association between East Asian lineage and acquired resistance to fluoroquinolones, although the number of cases analyzed was too small to support multivariable analysis to identify potential confounders.

The association between *M. bovis* and acquired resistance to both isoniazid and rifamycins may be partly attributable to its inherent resistance to pyrazinamide, a drug included in standard four-drug regimens used to treat drug-susceptible tuberculosis [17] because of its sterilizing properties and its ability to kill dormant, non-replicating bacilli [18], [19]. However, our results suggest that resistance to pyrazinamide may not entirely explain the association between *M. bovis* and acquired resistance, as initial resistance to pyrazinamide did not predict acquired resistance to isoniazid or rifampin in our analysis. Issues with false positivity associated with standard methods for testing pyrazinamide susceptibility [20], [21] may have contributed to this lack of an association. However, only about half of the cases included in our analyses that had initial resistance to pyrazinamide were caused by *M. bovis*, and when *M. bovis* cases were excluded from analysis, no association was observed between resistance to pyrazinamide at initial DST and acquired resistance to either isoniazid or rifamycins. These results suggest that *M. bovis* may have attributes other than resistance to pyrazinamide that increase the likelihood of its acquiring resistance to other drugs.

The finding that East Asian lineage is associated with acquired resistance to fluoroquinolones is consistent with the numerous studies reporting associations between East Asian lineage or the Beijing genotype with drug resistance [9]–[13]. Furthermore, an association between the Beijing genotype and primary fluoroquinolone resistance has been reported [22]. A potential biological mechanism that could explain associations between this lineage and both initial and acquired drug resistance has been suggested by *in vitro* studies reporting that East Asian strains have an elevated basal mutation rate compared to East African-Indian and Euro-American strains [23], [24], and are therefore more likely to develop mutations leading to drug resistance. Finally, our observation that acquired resistance to fluoroquinolones was associated with East Asian lineage but not to region of origin suggests that the observed association is not attributable to host factors among persons from regions where the East Asian lineage is common [8].

Our observation that initial resistance to isoniazid is associated with acquired resistance to rifamycins is consistent with other studies that have shown initial resistance to one or more drugs to be a strong risk factor for additional acquired resistance [25]. Surprisingly, we did not observe strong evidence for an association between initial resistance to rifamycins and acquired resistance to isoniazid. The lack of association could be attributable to the small number of cases with initial rifamycin resistance in the absence of initial isoniazid resistance. Out of 3,692 cases with initial susceptibility to isoniazid and a documented initial DST result for at least one rifamycin, only 20 (0.5%) were resistant to rifamycins. In contrast, 328 (8.2%) of the 3,995 cases with initial susceptibility to rifamycins and a documented initial DST result for isoniazid were resistant to isoniazid.

The association between HIV infection and acquired resistance to rifamycins may be attributable to interactions between rifamycins and antiretroviral drugs used to treat HIV [26], and is consistent with previous studies that observed acquired resistance to both rifampin and rifapentine in patients being treated for TB-HIV [27], [28]. However, we could not test this hypothesis as NTSS does not collect information on antiretroviral therapy. The basis of the negative association between unknown HIV status and acquired resistance to isoniazid is unknown, but may reflect an association not with HIV status but with particular tuberculosis control programs that did not report HIV status of patients during the time period of this analysis [29].

Finally, we observed an independent association between self-administered therapy and acquired resistance to rifamycins, reinforcing the rationale behind using directly observed therapy (DOT) to promote completion of treatment and discourage acquisition of drug resistance [17], [30]. This contrasts with results of a recent meta-analysis of prospective observational studies and controlled trials, which found no statistically significant pooled risk difference for DOT versus self-administered therapy based on two included studies reporting acquired drug resistance as an outcome [31]. In their discussion, the authors suggest that retrospective studies reporting an association between poor treatment outcomes and self-administered therapy as compared to DOT have typically been unable to distinguish between the effect of direct observation itself and that of the programmatic improvements that accompanied the establishment of DOT as the standard of care. We believe our findings do not reflect this limitation because the current U.S. national TB elimination strategy, including DOT, was implemented during the 1990s, and important programmatic improvements are not likely to have been major factors during 2004–2011.

Our study was subject to several important limitations. First, initial DST for first-line drugs is routine, but repeated DST is discretionary in the United States. Therefore, the cases included in our analysis represented a small proportion of genotyped tuberculosis cases in NTSS, and are not representative of the general tuberculosis patient population in the United States. Because clinicians are more likely to repeat DSTs for patients because of clinical indications such as apparently ineffective treatment, the prevalence of acquired resistance among cases included in our analysis likely overestimates the prevalence in the general tuberculosis patient population. Furthermore, because DST for second-line drugs is also discretionary, very few genotyped cases had both initial and final DST results for injectables and fluoroquinolones. The small number of cases with acquired resistance to these two drug classes prevented us from analyzing the association between acquired resistance and *M. bovis*, and may have contributed to the lack of an observed association between lineage and acquired resistance to injectables. In addition, because of the small number of cases, we were unable to perform multivariable analyses, although clinical and sociodemographic factors associated with acquired resistance to both injectables and fluoroquinolones have previously been reported [32]. Finally, the small number of cases with acquired resistance to any drug makes overinterpretation of results possible.

Our study was also limited by the data available through NTSS and NTGS. Because only DST results for the first and last isolate tested are reported to NTSS, we could not include time to acquisition of drug resistance in our analysis. In addition, we could not include treatment regimen in our analysis because only the drugs in the initial regimen are reported to NTSS. Finally, while drug availability in individual patients may influence risk of acquired resistance [33], no data on serum drug concentrations or other pharmacokinetic measurements were available in NTSS that would enable us to consider this factor in our analysis.

Based on the data available in NTGS, we did not have genotyping results for both initial and final isolates and therefore could not confirm that the same strain was tested at initial and final DSTs. It is therefore possible that some cases were misclassified as having acquired resistance when in fact the change in DST result was attributable to a mixed-strain infection or re-infection during treatment, although the risk for either is generally thought to be low in low-incidence settings such as the United States [34], [35]. In addition, although NTGS has been performing routine genotyping on culture-positive cases since 2004, not all states started utilizing the service comprehensively at the same time, and the proportion of culture-positive cases with available genotypes ranged from 52% in 2004 to 89% in 2010. Because some states selectively genotyped cases in earlier years, cases with certain characteristics, such as being associated with outbreaks, may be overrepresented in our sample.

In conclusion, among patients reported in the U.S. surveillance system with both initial and final DST results, *M. bovis* was independently associated with acquired resistance to isoniazid and rifamycins, and East Asian lineage may be associated with acquired resistance to fluoroquinolones. These results suggest that knowledge of risk factors for *M. bovis* infection such as Hispanic ethnicity, age <15 years, and extrapulmonary disease [36] may be useful for identifying patients with increased risk for acquired isoniazid and rifamycin resistance. In addition, patients from countries in East and Southeast Asia, where the East Asian lineage is common, may be at increased risk for acquired resistance to fluoroquinolones [8]. However, given the limitations of U.S. surveillance data, prospective clinical studies are needed to confirm the clinical significance of these findings, including whether rapid genotyping of isolates at the outset of treatment may benefit patient management.
